# Aplastic anemia in adult and pediatric hematology

**DOI:** 10.1097/HS9.0000000000000261

**Published:** 2019-06-30

**Authors:** Charlotte Niemeyer

**Affiliations:** University Children's Hospital, Freiburg, Germany

**Learning goals**

- To recognize the clinical implications of our current understanding of SAA pathophysiology
- To be able to identify an appropriate treatment strategy—immunosuppressive therapy, stem cell stimulation, HSCT—for individual patients with SAA
- To understand the significance of somatic mutations detected in bone marrow of patients with SAA and hypoplastic MDS.

## Introduction

Immune-mediated severe aplastic anemia (SAA) typically presents with rapidly decreasing blood counts of all lineages and a profoundly hypocellular bone marrow. Much effort has been invested in characterizing its immune signature targeting hematopoietic stem and progenitor cells. In this education session Neal S. Young M.D. will summarize our current understanding of the SAA immune phenotype and outline principles of standard therapy with immunosuppression and more recently stem cell stimulation. Andrea Bacigalupo M.D. will discuss current indications for allogeneic hematopoietic stem cell transplantation (HSCT) in SAA and develop future perspectives of upfront alternative donor grafts. It has long been appreciated that abnormal clones can expand in SAA bone marrow. Judith Marsh M.D. will review the impact of the immune signature of SAA and hypoplastic myelodysplastic syndrome (MDS) on the development of somatic mutations and outline their clinical significance for classification, therapy, and outcome.

